# Implementing Trauma Informed Care in Human Services: An Ecological Scoping Review

**DOI:** 10.3390/bs12110431

**Published:** 2022-11-02

**Authors:** Daryl Mahon

**Affiliations:** Outcomes Matter, A63RK65 Wicklow, Ireland; outcomesmatter1@gmail.com or darylmahon@gmail.com

**Keywords:** trauma informed care, trauma informed practices, ACE’s, trauma responsiveness, implementation, organizational implementation, scoping review

## Abstract

Trauma and toxic stress are growing public health concerns with increasing risks to morbidity and mortality. Trauma informed care is an organizational response that challenges providers to adapt principled based approaches that seek to reduce adverse effects of care and support healing. However, there is a scarcity of empirical evidence on how trauma informed care is implemented in systems. A preferred reporting items for systematic reviews and meta-analysis-compliant scoping review based on Arksey, and O’Malley’s five steps model was conducted. Four databases, PubMed, Scopus, Embase and PsychINFO were searched for English articles published since 2000. Studies were included if they reported on trauma informed care delivered by services that support adults and there was some reference to implementation or organizational implications. Of 1099 articles retrieved, 22 met the inclusion criteria. Findings suggest that trauma informed care is being implemented in a range of human services, including at the city/state level. While implementation research is still at an early stage in this field, the findings elucidate several challenges when implementing this approach across systems of care. An ecological lens is used to present findings at the macro, mezzo, and micro level, and these are further discussed with reference to practice, policy, and research.

## 1. Introduction

There is an increasing recognition that many people accessing human services may have had prior traumatic experiences, and that a paradigm shift in how service delivery is organized may need to occur to be responsive to these experiences [1,2,3,4]. The findings from the Adverse Childhood Experiences (ACEs) seminal research study are of importance and support the need for trauma informed care (TIC). This large retrospective study identified a correlation between childhood trauma and subsequent health in a large sample of 17,000 adults. Findings highlighted that ACEs impact all aspects of a person’s wellbeing, and possibly reduce life expectancy [5,6,7]. Single incidence trauma is suggested to have a prevalence of approximately 70%, with 30.5% of people experiencing four or more such incidence [8,9] Traumatic experiences are said to have a cumulative impact on the individual, with more adverse experiences increasing the risk of ill health.

Notwithstanding the importance of adverse childhood experiences on health outcomes, several limitations are apparent. For example, the initial study was conducted with largely Western, white middle class demographics [5].

ACEs were researched at the population level, meaning individual experiences were absent [10]. Furthermore, variables that are said to impact trauma experiences, such as socio-economic, cultural, and environmental factors were missing from the ACE checklist [11]. Thus, while the ACE research has orientated the field towards the impact of trauma, it provides for a narrow definition and conceptualisation, which means other potential traumatic experiences such as racism, discrimination and other important social determinants of health may not be considered.

Within human services, some cohorts accessing supports present with higher trauma prevalence than the general population, with rates as high as 88% reported in some settings [12]. Childhood trauma is also linked to increased social service costs [13,14]. In fact, in America, trauma is considered to cost over $3 billion annually in days missed from employment. Similarly, in Europe, Adverse Childhood Experiences are thought to have an annual cost of $81 billion [15,16].

It is not just service users accessing supports who have experienced traumatic incidences, employees’ working in human services are also reported to have high prevalence of trauma, which may exacerbate secondary traumatic experiences [17]. At the same time, employee personal trauma, and the impact of vicarious trauma may be compounded by the environment in which the employee works [18,19]. For example, the climate and culture within the organisation, practices, as well as lack of supportive supervision and leadership may compound these trauma experiences [19,20]. Bloom [21] describes the impact of organizational stress as permeating across the entire system of an organization impacting all stakeholders, and levels of service delivery, care, and outcomes. As such, healthcare systems and policy makers are increasingly recognizing the need to be more trauma responsive.

There are several definitions of trauma informed care put forward [4,22,23,24], with some of these literatures going beyond definitions and providing value-based principles to help inform practice. Trauma informed care (TIC) is one way that organizations can respond to the needs of service users and employees. TIC is a universal method of service delivery that acknowledges that many people accessing services, and employees working in services, may have experienced traumas, it also realizes that how service settings are constructed and delivered can help support individuals in their healing, or hinder people through re-traumatization [3,4,21]. On this point, trauma informed care as a universal organizational approach assumes that many people accessing services and employees may have potentially experienced trauma. Therefore, TIC embraces this system wide approach to reduce the possibility of retraumatizing people during their interactions with the organization [2,4]. In comparison, trauma specific care is based on the treatment of a person’s trauma, using trauma focused therapies to alleviate trauma symptoms [25,26]. A working definition of TIC contents that it is

“*A program, organization, or system that is trauma-informed realizes the widespread impact of trauma and understands potential paths for recovery; recognizes the signs and symptoms of trauma in clients, families, staff, and others involved with the system; and responds by fully integrating knowledge about trauma into policies, procedures, and practices, and seeks to actively resist re-traumatization*”

[2] (p. 9).

Trauma informed care involves a paradigm shift in how services are delivered, and implementation science informs us that implementing evidence-based interventions through systemic culture change can be a difficult process [27,28,29,30].

Whilst the implementation of TIC has been the topic of several evidence syntheses previously [31,32,33] these have focused exclusively on child and adolescent services.

Yet, the implementation and practice of trauma informed approaches in child and adolescent services may entail differences based on developmental factors that do not generalize into adult services. For example, child welfare services offering trauma approaches with young children at early stages of cognitive development may not be able to implement choice and collaboration in a trauma informed way, or cultural identity may be at very early stages of socialization meaning these principles and the principle of peer support may not be used in organizational approaches. As such, there is a gap within the literature pertaining to what we know about the implementation of TIC in human services or communities working mainly with adults, and this scoping review can aid our understanding and support future research and practice. Human services in this paper are defined as those health and social care settings offering supports to individuals across psychosocial domains, for example, mental health, homelessness, substance use and disability.

## 2. Materials & Methods

Arksey & O’Malley’s [34] five step scoping review was conducted on the implementation of trauma informed care in organizations. The scoping review is ideally placed to answer this question, as opposed to other types of evidence synthesis with a more limited focus [35,36,37]. This scoping review will report on the characteristics of the included studies through an ecological framework, informed by a thematic and numerical narrative analysis [34,38]. The ecological framework is based on the work of Bronfenbrenner [39] and examines the complex interactions between the individual, relationships, community, and other sociocultural factors in the wider environment. These interplays are conceptualised across a micro, meso, and macro system. This review is consistent with the Preferred Reporting Items for Systematic reviews and Meta-Analyses extension for Scoping Reviews (PRISMA-ScR) Checklist [40]. To provide for a wider range, the scoping review proposes a broad and well-defined question that clearly defines the constructs, concepts and type and scope of outcomes to be considered. The research question was developed using the population, concept, and context (PCC) framework proposed by [41]. To facilitate a broad examination of the literature the following question is posed:

*RQ1: What can we learn about the practice and implementation of trauma informed care in organizations or communities*

An electronic database search for peer reviewed and grey literature was conducted in Scopus, PubMed, PsycINFO and Embase using the following search strategy (title-abstract-keywords (trauma AND informed *) OR title-abstract-keywords (ace *) AND title-abstract-keywords (implementation *) AND NOT title-abstract-keywords (children *). Titles, abstracts, and keywords were searched for studies that addressed the research question. In addition, the first 10 pages of Google Scholar and Google were searched, and the bibliography of relevant studies searched for further references. Inclusion/exclusion criteria (Table 1) were set posterior using an iterative process after an initial trialing of the search strategy was conducted. There were limiters applied on dates of included studies, 2000 to 2022. English language articles reporting on primary data in the context of trauma informed care, and which had some mention of implementation or organizational considerations in human services offering supports to adults were included. The search was initiated on the 15th of July and the cut off for any further articles was the 30th of July 2022.

**Figure 1:**
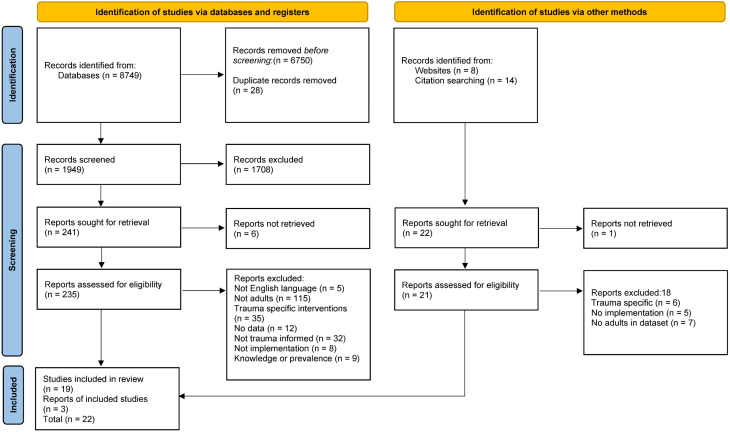
The flow of information through the different phases of the scoping review and the rationale for inclusion/exclusion.

A total of 1099 articles were identified from databases, with four further articles identified from bibliographies. All articles were downloaded into Mendeley software for appraising by the author, with those not meeting the study criteria excluded. A total of (22) articles met the inclusion criteria for this review. The PRISMA-ScR flowchart (Figure 1) outlines the process involved in this study selection [42].

Data was extracted by the author and placed into excel with pre-determined headings (Table 2) that were broadly consistent with Gerrard’s Matrix Method [43]. The headings included, author and study year, location study was conducted, article type, sample size, target group, study design, intervention being investigated, outcomes, outcome measures used. Articles were uploaded to NVivo, and codes were developed based on the literature and charted data, and then assimilated into broader abstract themes, which are discussed below using an ecological framework of macro, meso and micro level of analysis.

## 3. Results

In total (N = 22) studies met the inclusion criteria for this scoping review, all were conducted in high income countries, mostly America (N = 17), Canada (N = 3), United Kingdom (N = 1) and Australia (N = 1). The included studies were peer reviewed journal articles (N = 17) doctoral dissertations (N = 3), and reports in practice-based settings, (N = 2), with a sample range of (N = 4) to (N = 760), total (N = 2273). Case studies were reported in (N = 5), mixed methods (N = 6), qualitative (N = 7), quantitative (N = 4), and longitudinal (N = 1), and (N = 4) were evaluations.

The target groups of the studies included substance use (N = 5), disability (N = 2) whole city/community level (N = 2), homelessness (N = 1) with the remaining studies occurring across a variety of other human services (N = 12). A battery of outcome measures and fidelity measures was reported as being used in (N = 12) papers. Harris and Fallot’s (2001a) conception of trauma informed care was the model most cited (N = 6), and SAMSHA (N = 2), with the remainder of studies using undefined or self-developed models of TIC. Overall, the literature exposes a lot of heterogeneity with regard to where and how trauma informed care is being implemented, and the range of outcomes that are being reported on in the literature and practice-based settings.

### 3.1. Macro System

The results are presented using a multilevel framework based on an ecological analysis using macro, meso and micro domains. In this analysis, macro factors refer to the wider structures outside of the organisation, socioeconomic, commissioning, and regulatory bodies, inter-agency approaches and community factors. The wider ecology where organizations are embedded emerged as important to consider with regard to successful implementation of TIC. Financial resources were identified as integral to implementing TIC and ongoing monitoring or evaluation, this financial capital was generally sourced through grants at city or state level, or from charitable trusts, and the lack of financial capacity was experienced as a barrier to implementation [7,11,14,18]. This is consistent with prior research suggesting that lack of financial capacity can act as a barrier to TIC implementation [66,67]. Where health insurance is willing to reimburse organizations for screening, this was experienced as an enabler [3]. With regard to monitoring and evaluation at a macro level, Matlin and colleagues [11] used a community participatory implementation strategy approach to develop a shared vision of TIC through the development of a logic model that was used to inform an evaluation and to collect data at different points throughout the implementation in the community and to make adaptations to the implementation process. Two other studies described developing strategic partnerships with local universities to support the implementation process through research, planning, providing training and evaluations [9,11]. These two studies reported on the development of task forces/committees to drive the implementation of TIC across communities. The committees were made up of key stakeholders at various levels from the community and were responsible for planning, resourcing, and evaluations [9,11] in doing so, these committees acted as implementation enablers as they could identify and remove possible barriers and facilitate a range of fundings and resourcing to promote implementation at different levels. These results would reflect that of the wider implementation literature with regard to establishing committees to guide implementation [27,28] and the trauma informed literature specifically where Mahon [3] describes the use of a Trauma Oversight Group (TOG) to resource and guide implementation.

The establishment of interagency collaboration is integral to TIC, and it was identified as important to the implementation process in [3,11,21,22]. When considered within another study seeking to make the State of California ACE aware, it underscores the importance of using the wider ecology as a resource for referrals. In the California study, Nanson [3] found that those services in rural areas often had less resources and referral options, which itself has implications regarding whether it is appropriate to offer screening if there are no follow up supports. However, this review exposes that there is little empirical research with regard to describing TIC at the system level, its operationalization across systems, and the processes, procedures, and policy to support inter-agency collaboration between organizations.

The legislative and regulatory environment where organizations and communities provide services can equally act as a barrier or facilitator to implementation. The role of regulatory bodies mandating TIC practices was experienced as a barrier in two studies [2,3]. While nurses found the regulation of TIC practices in general to add to an already busy workload restrictive [2], some practitioners found mandating a particular screening tool to be restrictive to practices, and without the desired evidence base [3]. From an implementation perspective, we can understand that this may act as a barrier to uptake when we consider how compatible the intervention is with practitioners’ values, and the perceived relative advantage it offers them [68].

### 3.2. Meso System

The meso level of analysis is used to describe the organizational factors such as leadership, training and education, involvement of stakeholders and the general governance of the organization. Leadership was discussed as an integral component of implementation across several studies [47,52,54,55,58,63,64,65]. More specifically, the implementation of TIC helped change leadership and management practices to embrace principles of trauma informed care, particularly with regard to employees having more choice, being empowered and collaborating in a shared decision making process [57,63,64] this was often experienced as a flatting of the organizational hierarchy [56,62,64], which in turn helped operationalize the principle of trust [64]. It was assumed that these changes in the organizational leadership approach were responsible for improved organizational climate and satisfaction of employees [48,60,61,62,63,65]. However, not all employees were satisfied with this change in leadership style, the impact of flatting the hierarchy was described in a negative manner by those employees with less experience, whereby attempts to promote independence in decision making was not experienced as positive [64].

These findings indicate that this type of leadership is effective and conductive of positive outcomes, however, it may be that a more situational leadership style will benefit others, especially where experience and expertise is still developing. Outside of flatting the hierarchy and operationalizing some of the principles of TIC it is unclear what type of leadership is being exercised in the studies in this review, or if one type of leadership is better suited to TIC organizations. Whilst sharing power in this way was generally experienced positively, a more directive approach was needed when programme drift was occurring to maintain fidelity to TIC [64]. Buy in from senior leaders was identified as being integral and that senior leaders needed to champion TIC [46,55,57,65], These findings around leadership are consistent with the wider implementation science literature whereby leadership is seen as a key enabler of implementation [69,70], and the trauma informed care literature specifically [31,46,64]. A collegial approach is essential to implement trauma informed care. Two studies [46,54] describe using what can be termed communities of practices to help support capacity building and networking for the purpose of implementation within their respective communities. These communities of practice acted as enablers to implementation through shared learning environments.

Similarly, the involvement of wider stakeholders through co-production is suggested to be a key implementation domain [2]. The involvement of end beneficiaries in the planning, design and delivery of services was discussed in [52,54,56,57]. Changes to hiring practices were noted also, specifically seeking to hire more racially diverse leadership positions [64], and hiring employees with characteristics more aligned to TIC [57]. From a policy perspective there was very little in the included studies, only three of which referred to making changes to organizational policy to support implementation [52,53,63].

Based on this review, we can conclude that there are still questions to be answered with regard to the role of policy in conceptualizing, operationalizing, and implementing TIC within and across systems of care. Previous research found that a lack of clear policy can be a significant barrier to implementing TIC [70,71]. Various other studies in this review utilized different trauma informed care measures, or measures to track implementation in general at the organizational level [44,47,48,49,52,53,55,56], which may indicate that those implementing TIC understand the complexities involved in transformational change. Some of the measures in the studies included in this review were developed by the researchers or adapted from existing measures indicating a scarcity of methods to measure implementation, and the various intended outcomes in their entirety.

It is recommended that an introductory training in trauma informed care precedes implementation [2,3,4]. In this review, training was discussed in [45,46,50,52,54,56,57,59,63,64,65]. More specifically, TIC training should be provided to all employees from administrators to clinicians [46,50,54,57,59,65], and while this was generally done by individual organizations, train the trainer models were used to help disseminate and implement TIC into the wider systems also [54,57]. Importantly, foundational knowledge developed from training was found to act as an enabler to the uptake of TIC through self-efficacy and impacting beliefs and attitudes [55].

Two studies [46,65] suggest that incorporating TIC into university degree curriculum should be considered thereby preparing practitioners before they get into communities and organizations, making implementation a more seamless process through the uptake of an intervention. On this note, trauma informed care is often conceptualised differently dependent on where it is being practiced, and the findings from this review illustrate different models and curriculum used across the various contexts. This lack of standardisation does pose challenges for establishing an evidence base as the outcomes from one model cannot be assumed to be the same as another model/. However, from an implementation perspective, it could be that certain models do fit better to some contexts and populations than others. Yet, no rationale was provided as to why organisations in this review would choose one model over another, nor does the extant literature currently address this issue in any meaningful way. This does pose then question then, what are the active/specific ingredients of TIC.

While several studies described universal screening occurring within their organisation, resource allocation, time constraints, and utility of screening instruments meant that screening was sometimes viewed negatively and had the potential to become a barrier to implementation [46,47,57,58]. In addition the need for training competencies in screening interventions [46,58] was identified as a further barrier to the uptake of screening.

### 3.3. Micro System

The micro level analysis in the ecological framework focuses on individual practitioners, and service users related outcomes and any other individual level variables. Client outcomes were reported in various studies [44,49,52,53,59,61]. One study that linked TIC to a range of psychosocial outcomes is noteworthy.

Shier and Turpin [44] found that a TIC environment characterised by the principles set out in [2] were correlated with service users intrapersonal functioning, as well as a reduction in dual diagnosis symptoms. Despite this, the review does expose that there is a scarcity of client outcomes in the literature, the field needs to start examining methods of monitoring and measuring these clinical outcomes, in addition to demonstrating how TIC organisation are impacting on other important service user outcomes.

Psychoeducation of trauma experience is an essential intervention and discussed as a core component of TIC in [52,59,64]. However, several barriers to using psychoeducation emerged, for example, employees are often reluctant to discuss trauma with service users because of fear of re-traumatisation [45,58]. It was also recognised that employees often have their own trauma, and without adequate supports and training some may be hesitant to enquire into service user’s experience of trauma [46,63,65].

Trauma informed care training was described in several studies as an enabler by helping to build the knowledge, attitudes, and beliefs of practitioners. Pre-post studies [44,52,54,62,63] that assessed knowledge, beliefs and attitudes demonstrated that training could impact on these employee variables and can help build employee commitment to TIC. Self-rated competency in TIC was a predictor of TIC practices [58], considering some of the issues with regard to employees being hesitant about screening and asking service users about their trauma experiences, this underscores the need to provide training to develop these competencies in employees. However, the extent that these variables impacted on either implementation or service user outcomes in this review is largely unknown.

Several studies spoke about the importance of providing supports for employees for self-care purposes and to mitigate against vicarious trauma reactions [46,50,52,57,59,65]. However, some studies identified a gap in this area with more of a need to be responsive to employee burnout and re-traumatization [57]. In general, employee satisfaction was rated favorably after the implementation of TIC [51,52,61,62] and employees experienced many of the trauma informed principles and practices by leaders in their respective organizations [48,63,64,65]. Yet, in some studies, the safety of employees was thought to be compromised due to the implementation of TIC and changes in practices [60,61].

Safety was the principle most often discussed, however, safety often meant different things in different contexts, and as such, the lens safety is viewed through is contextual both for service users, and professionals. For example, implementing TIC was considered as possibly removing the sense of safety that institutionalization provided in the context of routine and structure for people living in residential disability settings [61]. While both physical and emotional safety were suggested to be cultivated by providing housing to domestic abuse survivors in safe neighborhoods that survivors choose. Other barriers to implementation included employee resistance to change [45,46,57,60,64] and time constraints for employees to implement these new practices, including screening [45,46,57,58,65] Interestingly, Chalakani [51] found that resistance to implementing new TIC practices could be identified and mitigated against by using organizational data and employee feedback, for example through surveys or employee exit interviews.

### 3.4. Quality of Studies

Although it is generally not the aim of the scoping review to assess the quality of included studies [34], there is still benefit in providing a summary of the methods used in individual studies. In this review there is a lot of heterogeneity, and no replication research, however this needs to be viewed in the context of TIC implementation research still largely being in its infancy.

At the same time, many of the studies were qualitative [45,50,53,57,58,64,65], with small datasets [45,50,51,59,60] or case studies and evaluations specific to one organizational context [46,49,50,51,61]. While providing important information the transferability of these studies outside of their environment may be limited, also there are no experimental studies included which does mean that correlations presented are open to interpretation, and generalizability and transferability of findings may be limited. Although several dissertations and reports are without peer review, they do provide useful information from the grey literature.

Despite some of these methodological limitations, other studies using mixed methods to triangulate data [47,54,63], those with samples of multiple sites or using whole communities/cities [44,46,48,49,54,57,63] and longitudinal studies with multiple data points [52,54] lend themselves well to the topic of implementation and also tended to have larger datasets. In addition, pre-post studies that explored a range of organizational factors, employee satisfaction, knowledge and attitudes and training within the context of implementation can help us understand some of the intricacies of what is becoming a more complex area of research and practice.

### 3.5. Review of Outcome Measures

All together there were 25 measures used across the studies in this review (Table 3). The reliability was reported for 20 of the measures as adequate to high, only nine measures provided information on validity, all of which support strong construct validity. While in general where the psychometric properties of the included measures are reported on, these are adequate, however, not all studies report the psychometric properties and 4 of these do not report any reliability. Finally, the lack of experimental designs does limit generalizability of the findings from some of the outcomes where measures were used, as well as potential biases not being accounted for. Regarding specific measures related to trauma, or trauma implementation in the organization, 12 measures were used, with the reminder assessing other aspects of organizational development or beliefs, attitudes, and satisfaction. Some other measures assessed aspects of trauma informed care, but the measures were not specific to trauma, with one of these reporting data on clinical symptoms such as addiction and mental health.

## 4. Discussion

This scoping review sought to identify how trauma informed care is being practiced and implemented in organizations or communities where adults are provided with services, analyzed under an ecological lens. While trauma informed care may feel intuitively right, it is currently still more a hopeful ideal than an empirical reality, in the context of this review, at least. However, there is still much to be hopeful about, as research on implementation is relatively new and exposing areas for fruitful investigation.

The results from the studies included in this scoping review demonstrate lots of heterogeneity, while also providing insight into the large number of fields of practice that TIC is being provided in, indicating its trans-theoretical reach. While collectively this review included most of the relevant aspects of TIC, there was variation among studies with regard to the different principles and components of TIC studied, perhaps for these reasons this review provides limited evidence of service user and employee outcomes. Despite these issues, there is still lots we can learn from the findings related to satisfaction, and outcomes related to training, in addition to some organizational culture, climate and leadership domains and the relationship with implementation. In their systematic review, focused on services provided to young people, Hanson, Rochelle and Lang [72] contend that the least likely components of trauma-informed care to be implemented in their studies were those measuring staff proficiency, a defined leadership position, addressing secondary traumatic stress and written policies that addressed trauma. While many of these areas have been addressed to some extent in this review (although much more is needed), neither peer support nor multicultural principles were discussed. This could be due to only two studies focusing on SAMSHA [4] model, with more focused-on Harris and Fallot (2001a) model, which does not include these two areas. Considering the prevalence of trauma experienced by those of different ethnic identities, cultural responsiveness should be an essential aspect of trauma informed care.

For example, systematic review and meta-analyses have found elevated levels of trauma among refugees [73,74].

Other ethnic and minoritized communities often have higher prevalence of trauma and use social service more frequently, in addition to employees of social services having high rates of trauma themselves [17], some of whom presumably also have ethnic identities. This underscores that for both service users and employees’ cultural considerations are important, and while more research is needed, some studies have provided insights into how providers can become more culturally responsive [75,76], and by demonstrating cultural humility [3,77]. While meta-analyses provide evidence of peer support across mental health and substance use [78,79,80] there is a paucity of research in the extant literature as it relates to peer support from a trauma perspective specifically. Although two papers do provide initial insights into the role peers can play in supporting people with trauma [81,82] much more research needs to be done in this arena, if peer support is to be included in organizational responses as part of TIC.

Drawing on the implementation science literature is another way that can assist us in making sense of the findings in this review. For example, while training in TIC was experienced as an enabler and provided benefits to participants with regard to knowledge, beliefs and attitudes, implementation research informs us that providing information and training without other organizational supports at the macro and mezzo level is unlikely to change practices or culture [27,28,83] While some studies in this review discussed organizational change to support training, others did not, and even those studies where supports were in place, there was a lack of assessment of how the principles of TIC were being implemented, which has implications for fidelity to TIC.

Although several studies did report on operationalizing TIC with service users through shared decision making [44,49,50,52]. While many studies included fidelity measures of organizational implementation, these measures do not provide a standardized method of assessing fidelity to TIC principles.

In this review, one study assessed TIC practice based on perceived competency, with those employees rating themselves as more competent in TIC being more likely to practice TIC, this key enabler should be considered by organizations planning to train employees and implement TIC going forward. Indeed, a systematic review that focused on employee training in TIC found that personal attributes of employees is a facilitator of implementation [84]. This has implications from an implementation science perspective as uptake of an intervention such as TIC is enabled if employees believe that they are more knowledgeable, skillful, or competent in the delivery of an intervention [85,86,87]. For example, in some of the studies in this review, employees were often reluctant to complete screening or to ask service users about prior trauma experiences due to not feeling prepared to do so. Screening is an important part of TIC, however, may screening measures in this field are not suitable, have the required evidence base or psychometric properties, and this can act as a barrier to uptake [68] by employees and organizations. Developing the correct type of screening instrument is one way to encourage uptake of screening. The wider literature suggests that measures need to have utility and brevity for practitioners to use them routinely. An accurate screening measure of trauma, based on the classification of complex trauma set out by the World Health Organisation [88] may be one way to achieve these aims.

Leadership and management are an integral component of implementation. In trauma informed care implementation literature [2,3,4,21], leadership and management are discussed as key implementation domains. In this review, leadership and management were mainly discussed with regard to operationalizing the principles of TIC and with employee satisfaction outcomes and organizational climate. Leadership was associated with these outcomes by flatting the hierarch and involving employees in a process of shared decision making. Leadership styles were not discussed and as such, whether one leadership approach is better than another in TIC organizations remain unanswered. On this note, Mahon [3] operationalizes TIC through Servant Leadership, as this leadership philosophy involves reducing hierarchies through the redistribution of power, which in turn may help develop a climate conducive to not re-traumatizing stakeholders.

While training is an integral first step in TIC [2,4], follow up supports such as refresher training, coaching, supervision, and feedback are also integral to successful implementation [3,89,90] On this note, there was little by way of exploring how supervision, or leadership supported employees to operationalize TIC principles in this review, although championing and training were discussed as important to facilitators of implementing TIC.

There was limited information about how changes to policy supported overall implementation, within or across systems. Both leadership and policy are two key domains of TIC set out by [4], in addition to being important from an implementation science perspective in general [27,28], accordingly, they can act as key facilitators and enablers as demonstrated in this review. As such, identifying a defined leadership approach, and developing policies that specifically operationalize TIC and how it is to be implemented within and across systems is still needed.

Another important implementation domain discussed in the TIC literature is the monitoring and evaluation of TIC efforts. While several studies utilized implementation fidelity measures, this was largely a research endeavor as opposed to a practice development tool, and within these measures the psychometrics were not always reported on. A recent systematic review of measures used to assess implementing TIC came to similar conclusions with regard to the psychometric properties of measures, and the lack of outcomes associated with these instruments [91].

One exception is noteworthy and may be instructive for other implementation efforts. Matlin and colleagues used a logic model to inform a later evaluation and to use for multiple data collections during an implementation at the community level, meaning changes to the implementation process could be made based on information collected from key stakeholders. Of all the studies this is perhaps the most comprehensive with regard to implementation, and this monitoring and evaluation method is not only consisting with ideas from [3,4] but also consistent with ideas from the wider implementation science literature regarding feedback loops and making adaptations to scale out evidence-based practices [92].

On this later point, implementation science itself may be a limited lens through which to view implementation, especially as it relates to adaptations and transporting evidence-based interventions wholesale into complex systems where uptake may not always be welcome. In addition, implementation science has been critiqued as being linear, with too much of a focus on fidelity of interventions. Instead, Complexity science [92,93] is one way we can understand complex implementations involving adaptations. This may be of critical importance for TIC based on the findings of this review. Consider for example the findings that implementing TIC in some services may be compromising both service users and employee sense of safety, or that certain principles make practice more difficult [61,64] Thus, in these circumstances then, following fidelity to TIC principles, or the linear model of implementation science without making adaptations to fit contextual factors may act as a barrier to implementation and to reaching a sustainable level of organizational change. Again, these data do underscore the importance of developing the correct TIC principles depending on the type of service being offered, service users demographics and contextual factors, which may mean that the active/specific ingredients of TIC need to be established.

### 4.1. Implications for Practice, Policy, and Research

Based on the findings of this scoping review, some tentative recommendations are provided to support the further implementation of trauma informed care within systems, and for the research agenda going forward. Organizations or communities seeking to implement TIC should consider partnering with researchers with expertise in this area. Specifically, researchers can support implementation with pre-planning, setting up structures and committees, developing and providing training, and through ongoing consultation, monitoring and evaluation.

An initial training should be provided to all members of the system, where the system has multiple sites, or the organisation is large, then train the trainer models should be considered, with follow up supports such as coaching, supervision and refresher training. Training should also be provided to relevant employees on universal screening and psychoeducation, to limit the extent to which employees do not feel competent or fear re- traumatizing service users. Methods to assess how competent employees feel should be employed, as this can be a predictor of later TIC practices. Where screening is occurring, organizations should develop clear policies to support practitioners and service users, and policy on inter-agency referrals to trauma specific organizations.

Those implementing TIC may need to consider which model is best suited to their individual context, with regard to what principles to include, for example, how safety and choice fit into the context of a service, or if peer support is relevant. As such, drawing on complexity science may be one avenue to consider for organizations and systems of care to help fit trauma informed care to their individual context and to make relevant adaptations. Tracking implementation through a monitoring and evaluative system in real time would be an ideal way to do this, adding value beyond the exclusive use of TIC organizational fidelity measures.

On this note, the use of Action Research to help implement evidence-based practices in service reform and complex change may be one method for those on the ground to consider [94]. Using action research to surface challenges during the implementation of TIC in real time and delivering this data to implementation groups on the ground has been found to be successful in managing complex change in healthcare. This can be supported with other mechanisms to provide a systemic feedback loop at all levels of the organisation, which should be supported by policy positions. Where planning for implementing TIC is occurring, co-production processes should be employed which involve key stakeholders and beneficiaries, the composition of implementation committees should include service users. Co-production can extend to other service providers, especially as establishing an inter-agency approach is important, co-production may aid this process. Where stakeholders are regulatory bodies, policy mandates should be conscious of the findings in this review regarding how such regulations may impact on service providers practices, motivations, and resistance. The leadership and management of organizations have an integral role to play in implementation, both in terms of providing a vision, and creating a climate that resources practices, championing TIC and modelling TIC principles. These areas have shown in this review to be linked to employee outcomes such as satisfaction, and organizational climate. These outcomes are linked to leaders operationalizing the principles of TIC, and flatting the hierarchy through shared decision making, this may also help develop trust in organizations and employee commitment to TIC.

With regard to future research, the case study is the most used method in this review, and it has illustrated many of the processes inherent in implementing TIC. However, to further the evidence base for TIC, more experimental methods should be considered that focus on service user and employee outcomes linking these to the principles and practices of trauma informed care. More studies are needed that include peer support and cultural practices also. In addition, further longitudinal studies with multiple data collection points focusing on specific areas of interest across the ecology may provide important insights into specific challenges and facilitators during implementation. More research is also needed on screening measures and measures of organisation implementation, especially with regard to their psychometric properties and how they correlate with employee and service user outcomes. Using SAMSHAS four Rs as a theoretical lens to examine organizational implementation and how services prepare to realize, recognize, respond to, and resist re-traumatization of service users and employees should be considered.

### 4.2. Limitations

There are several limitations to this scoping review that should be considered when interpretating the results. Firstly, this scoping review was conducted with a single author and while a review protocol based on PRISMA guidelines was adhered to, it remains that a second author may have been beneficial to the research process. However, following the PRISMA protocol and the steps involved does help limit any potential researcher bias.

Secondly, the reliance on articles in the English language may mean that other important sources could have been missed, and while the review did include grey literature, it is very possible that other unknown sources have likewise been overlooked.

Thirdly, the sixth step of Arksey and O’Malley’s [34] scoping review, including key stakeholders may have provided a more rigorous search strategy and analysis had key stakeholders been included. Fourthly, this review has no experiential studies which does limit the extant that the findings can be generalized into other settings. In addition, not all measures used reported on the psychometric properties. Moreover, as the scoping review does not provide a critical appraisal of included studies, possible limitations of inherent bias is not accounted for in this review. Finally, most of the studies included in this review were conducted in America, and although many of the implications can be considered trans-theoretical, there may still be various idiosyncratic factors that operate at the healthcare system level due to funding of services, meaning extrapolating to other systems of care in different cultures may be problematic. This final point would seem to reinforce the idea of taking a more complexity science approach to identifying and making adaptations to TIC based on context as opposed to exclusively following a linear implementation science approach only focusing on fidelity.

### 4.3. Conclusions

There is an increasing proliferation of trauma informed care in the literature, this scoping review took possibly the first ecological lens to analyzing how practitioners and systems go about implementing this approach into the culture of services. While there is still some way to go before trauma informed care is an empirical reality, the literature is demonstrating promising findings across employee and service users’ outcomes, while also identifying challenges to implementation across the wider ecology. However, more research is needed and the findings and recommendation in this review should be considered as they relate to future practice, policy, and further research.

## Figures and Tables

**Table 1:** Inclusion/exclusion criteria.

PCC	Inclusion Criteria	Exclusion Criteria
Language	English	Any non-English article
Type of article	Journal articles peer reviewed with dataset Grey literature with dataset Reports or evaluations with dataset Dissertations with dataset	Review articles Case studies without data Editorials Policy documents Systematic review/meta-analyses
Population	Organizations or communities providing services to adults, or studies where services provided to adults make up part of the dataset	Where data is reporting on service providers exclusively working with children or adolescents or families
Context	Any organisation or community study where trauma informed care as a universal method of service delivery is the main aim	Medical trauma in hospitals or other settings.
Concept	Has some mention of employee, or service user experience of TIC in the context of implementation, or organizational considerations of TIC	Trauma specific psychotherapy interventions, or interventions described only as PTSD, diagnostic assessment, or prevalence articles Research reporting on knowledge, attitudes, or experience of TIC without analysis of implications for implementation/organizational considerations TIC training without implementation considerations for TIC in adult services

**Table 2:** Charted data illustrating the headings data were extracted under.

Study	Location	Article Type	Target Group	Sample Size	Study Design	Intervention or Research Question Being Studied	Outcome Measures	Outcomes
**Shier & Turpin (2022) [44]**	Canada	Journal article	Dual diagnosis in residential treatment	(N = 172)	Multisite quantitative survey repeated measures	To test an empirical model of the effects of a trauma-informed organizational environment on service user outcomes in the context of concurrent disorder treatment	GAINs-SS. Intrapersonal Social Outcomes Development Scale; The Trauma-Informed Organizational Environment Scale	Service users at this organization had high overall trauma-informed organizational experiences, where staff engaged in behaviours that supported the development of safety, trust, choice, collaboration, and empowerment within concurrent disorder treatment programs.
**Blazejewski (2021) [45]**	USA	Dissertation	Nurses’ attitudes and beliefs about implementing TIC in disability	(N = 15)	Qualitative interviews	Theory of Planned Behaviour on implementing TIC	N/A	Four themes: nurses feeling empowered to avoid inadvertent patient re- traumatization, enhanced empathy towards patients, uncertainty about referents’ use of trauma-informed care, and the essential importance of being equipped and prepared
**Nanson (2021) [46]**	USA	Dissertation	The State of California	(N = 31)	Case Study qualitative	ACE Aware Initiative	N/A	Various themes related to implementation facilitators and barriers
**Piper et al. (2021) [47]**	USA	Journal article	Stakeholders at service for HIV	(N = 94)	Mixed methods survey and interviews	Barriers and facilitators to implementing TIC	Survey adapted from Trauma-informed Organizational Toolkit	Results highlighted the availability of several trauma services, including psychotherapy and support groups, but also revealed the absence of provider training on how to respond to patient trauma needs. Identified gaps in TIC services included written safety and crisis prevention plans, patient education on traumatic stressors, and opportunities for creative expression. Providers and staff supported implementation of trauma support services and employee trainings but expressed several concerns including resource and skill deficiencies.
**Robey et al. (2021) [48]**	USA	Journal article	Various mental and human health services	(N = 760)	Quantitative survey	Understanding Staff- and System-Level Contextual Factors Relevant to Trauma-Informed Care Implementation	The Consolidated Framework for Implementation Research (CFIR)	The attributes of the individuals implementing TIC and the implementation climate of the organization played the most central roles. identified available resources and the strength and quality of the evidence underpinning the intervention as important contextual factors for TIC implementation.
**Yoon (2021) [49]**	Canada	Dissertation	Women’s substance use programmes	(N = 9) Agencies	Case Study	Client perception of care and staff perceived organizational support	Creating Cultures of Trauma-Informed Care Program Fidelity Scale Version 1.3 (CCTIC) Ontario Perception of Care –MentalHealth and Addictions OPOC-MHA Survey of Perceived Organizational Support Scale (SPOS) Creating Cultures of Trauma Informed Care (CCTIC) Fidelity Measure	Although the CCTIC has utility to inform program planning and implementation for trauma-informed services, it may be challenged as a tool to evaluate the extent to which program domains are associated with client perception of care and staff perceived support.
**Bradley et al. (2020) [50]**	United Kingdom	Report	Bridge Water Womens Centre	(N = 12)	Qualitative interviews and focus group	An evaluation of a woman’s TIC programme	N/A	The findings of this evaluation highlighted some areas of good practice within the implementation of trauma-informed practice. These included embedding trauma awareness within hiring practices and the prioritisation of staff training and development to ensure that staff can better recognise and respond to the needs of women with histories of trauma
**Chalakani (2020) [51]**	USA	Disertation	Leadership experience of resistance during implementation	(N = 4)	Case Study	Leadership experience of employee resistance to change during the implementation of trauma-informed care	N/A	A change management process, effective use of available staff satisfaction data, and improved communication can lessen the experience of resistance. Trauma-informed care is seen as a positive change that enhances the workplace. Thus, its implementation is an opportunity for leadership to engage the workforce and the organization’s clients
**Hales et al. (2019) [52]**	USA	Journal article	Residential substance use	(N = 70)	Longitudinal with three collection points	To operationalize the processes, an agency can take to become trauma informed and assesses the impact of a multiyear TIC implementation project on organizational climate, procedures, staff and resident satisfaction, and client retention in treatment.	Trauma-Informed Climate Scale (TICS); Trauma-Informed Organizational Self-Assessment; Staff satisfaction was assessed using a 24-item instrument created internally; Client satisfaction was assessed using a dichotomous (yes or no) 42-item instrument created by the agency that assessed client satisfaction at 30 days, 90 days, and at discharge; Client retention was operationalized as client-planned discharge status	Following TIC implementation, there were positive changes in each of the five outcomes assessed. Workplace satisfaction, climate, and procedures improved by moderate to large effect sizes, while client satisfaction and the number of planned discharges improved significantly.
**Kusmaul et al. (2019) [53]**	USA	Journal article	Various health and human agencies	(N = 26)	Qualitative interviews	To understand how clients experience trauma-informed care services and implementation challenges.	N/A	The results of the study suggest that clients’ experience of these concepts was shaped by the actions of other clients, and these experiences were either mitigated or hindered by actions of the agency employees. Agency policies either supported or enhanced their experiences as well. The results also suggest that it was challenging for agencies to provide for all of the trauma-informed care (TIC) concepts at the same time
**Matlin et al. (2019) [54]**	USA	Journal articles	A whole community approach to TIC	Various N depending on different methodologies	Mixed-methods, multi-level design to examine PTICC processes at various times over its two-year initial implementation phase	A Tripartite Population Health Model of Trauma-Informed Practice: initiative—Pottstown Trauma-Informed Community Connection (PTICC)	Trauma Training Survey; Trauma Training Survey; Community Partner Survey; Attitudes Related to Trauma-Informed Care; Trauma System Readiness Tool; Levels of Collaboration Scale	The results show that moving forward the community is well-positioned to establish stronger inter-agency and system supports for trauma-informed practice in the service system and in the broader community.
**Sundborg (2019) [55]**	USA	Journal article	Particpants working in human services variables that predict affective commitment to TIC including foundational knowledge, principal support, self-efficacy, and beliefs about trauma	(N = 118)	Cross-sectional survey	The variables that predict affective commitment to TIC including foundational knowledge, principal support, self-efficacy, and beliefs about trauma	Affective Commitment Subscale; measure of foundational knowledge about TIC was created for this study; The Beliefs about Trauma Scale was created for the current study; Self-efficacy was measured using the Readiness for Organizational Change Scale (ROC); Principal support was measured using a six-item subscale from the Organizational Change Recipients’ Beliefs Scale (OCRBS)	Altogether, the model explained 65% of the variance in affective commitment to TIC
**Unick et al. (2019) [56]**	USA	Journal article	The relationship of individual and agency characteristics to the level of organizational TIC	(N = 345)	Quantitative survey	The relationship of individual and agency characteristics to the level of organizational TIC	Level of organizational trauma-informed care was measured using the TICOMETER	Weak relationships between individual factors and TICOMETER scores and stronger associations for agency-level factors. These included agency type, time since last trauma training, and involvement of service users. These findings highlight the importance of robust cultural changes, service user involvement at all levels of the organization, flattening power differentials, and providing ongoing experiential training.
**Duby et al. (2018) [57]**	USA	Report	Agencies who received funding to implement TIC	(N = 69) (N = 6) agencies	Qualitative interviews	An implementation analysis of efforts pursued by organizations participating in a grant program	N/A	A range of outcomes reported on; Changing organizational culture; Training and hiring; Promoting staff self-care; Screening for early adversity and trauma; Delivering trauma-responsive services; Involving patients; Facilitators and barriers
**Bruce et al. (2018) [58]**	USA	Journal article	Medical employees working in trauma centres	(N = 147)	Qualitative interviews	To examine health care provider knowledge, attitudes, practices, competence, and perceived barriers to implementation of TIC.	N/A	All participants rated the following as significant barriers to providing basic TIC: time constraints, need of training, confusing information about TIC, and worry about re-traumatizing patients. Self-rated competence was the most consistent predictor of providers’ reported use of specific TIC practices
**Ward-Lasher et al. (2017) [59]**	USA	Journal article	Homeless women experiencing domestic violence in Housing First	(N = 7)	Case study mixed methods	How practitioners and administrators implement trauma-informed care in a Housing First program for IPV survivors	Secondary survey data from the agency	Trauma-informed care principles and the Housing First model were found to be complementary. The majority of clients in this program retained housing up to 3-months after services ended and increased their safety and knowledge of domestic violence. Combining Housing First with trauma-informed care may increase success for survivors of IPV.
**Isober & Edwards (2017) [60]**	Australia	Journal articles	Nurses implementing TIC	(N = 5)	Mixed method Case Study	A nursing workforce practice development process to implement Trauma Informed Care as an inpatient model of mental health nursing care	N/A	While there are differing strategies for implementation, there is scope for mental health nurses to take on Trauma Informed Care as a guiding philosophy, a model of care or a practice development project within all of their roles and settings in order to ensure that it has considered, relevant and meaningful implementation. The principles of Trauma Informed Care may also offer guidance for managing workforce stress and distress associated with practice change.
**Kessler & Isham (2017) [61]**	USA	Journal article	Day service for disabilities	(N = 37)	Mixed method evaluation	To provide an initial conceptualization and preliminary assessment of TIC within IDD services in order to understand its impact among individuals and staff	Trauma Informed Care Measure	Three major categories emerged from the qualitative data (making a difference, recognizing progress and compromising factors), illuminating staff satisfaction with work experiences, individuals’ progress, and factors that challenged fidelity to TIC.
**Hales et al. (2017) [62]**	USA	Journal article	Mental health substance use implementing TIC	(N = 168)	Pre-post survey	The impact of implementing TIC on the satisfaction of agency staff	Business Insight survey from Workplace Dynamics. Two trauma measures (not named)	Following the implementation of TIC, agency staff reported higher scores on all but one of the six satisfaction survey factors. TIC implementation is associated with increased staff satisfaction, and may positively influence organizational characteristics of significance to social service agencies.
**Damien et al. (2017) [63]**	USA	Journal article	Citywide implementation Government workers and nonprofit professionals	(N = 90)	Mixed methods	The impact of TIC training on later implementation	Safety Attitudes Questionnaire (SAQ); Professional Quality of Life (ProQoL)	Use of a mixed-methods approach provided a nuanced understanding of the impact of TIC training and suggested potential benefits of the training on organizational and provider-level factors associated with implementation of trauma-informed policies and practices
**Kessler (2016) [64]**	USA	Journal article	Employees working in disability service	(N = 20)	Qualitative interviews	This study explores staff understandings and perceptions within an innovative trauma-informed day program for individuals with Intellectual/developmental disabilities	N/A	Reasonable understandings of trauma and TIC, highlighting factors critical to the five principles of TIC. Differences were associated with duration of employment and the presence of specialized training. Challenges with TIC emerged at different system levels: individuals, staff, management and interorganizational.
**Kirst et al. (2016) [65]**	Canada	Journal article	Substance use and mental health facilitators and barriers to implementing trauma informed practices	(N = 19)	Qualitative interviews	To explore facilitators and barriers in implementing trauma-informed practices	N/A	Key facilitators included: organizational support, community partnerships, staff awareness of trauma, a safe environment, peer support, the quality of consumer-provider relationships, consumer, and provider readiness to change, and staff supports. Challenges included: provider reluctance to address trauma, lack of accessible services, limited funding for programs/services, and staff burnout

**Table 3:** Psychometric properties of included measures.

Study	Measure/s	Reliability	Validity
Shier & Turpin (2022) [44]	Global Appraisal of Individual Needs Short Screener (GAINs-SS)	Cronbach’s a measure, the reliability of the total GAINs-SS at intake was 0.83 and 0.87 at program completion.	The short scale maintains good sensitivity and specificity for predicting diagnostic impressions
	Intrapersonal Social Outcomes Development Scale.	Cronbach’s a measure for each measure ranged from 0.91 to 0.94,	Fit indices as shown in support the adoption of all five factors, which are shown to have overall strong construct validity for sample sizes under 500
	The Trauma-Informed Organizational Environment Scale	Cronbach’s ranged between 0.84 and 0.89 for each of the factors, with the total measure at 0.87,	Fit indices from this analysis, indicated strong construct validity for every individual factor, as well as the higher order factor
Piper et al. (2021) [47]	Survey adapted from Trauma-informed Organizational Toolkit	N/A	N/A
Robey et al. (2021) [48]	Adapted from The Consolidated Framework for Implementation Research (CFIR)	Internal consistency reliabilitywas acceptable to good staff 0.87 system 0.74	TIC measure was positively related to staff-reported system support for TIC with a medium-sized effect, supporting its construct validity
Yoon (2021) [49]	Creating Cultures of Trauma-Informed Care Program Fidelity Scale Version 1.3 (CCTIC)	N/A	Not formally validated
	Ontario Perception of Care –Mental Health and Addictions OPOC-MHA	Cronbach’s alpha > 0.80.	Construct validity exploratory factor analysis showed two factor structure
	Survey of Perceived Organizational Support Scale (SPOS)	Cronbach’s alpha 0.93	Principle components factor analysis showed strong factor loading on the main factor, which was perceived support factor.
Hales et al. (2019) [52]	Trauma-Informed Climate Scale (TICS)	Cronbach 0.91	Confirmatory factor analyses supporting the scale’s construct validity
	Trauma-Informed Organizational Self-Assessment.	Cronbach’s for the total scale was 0.99	N/A
	Staff satisfaction was assessed using a 24-item instrument created internally.	Cronbach’s for the total scale was 0.94	N/A
	Client satisfaction was assessed using a dichotomous (yes or no) 42-item instrument created by the agency	N/A	N/A
Matlin et al. (2019) [54]	Trauma Training Survey	Cronbach 0.74	N/A
	Attitudes Related to Trauma-Informed Care	Cronbach 0.80	N/A
	Trauma System Readiness Tool	Cronbach 0.95	N/A
	Levels of Collaboration Scale	N/A	N/A
Sundborg (2019) [55]	Affective Commitment Subscale Measure created for this study.	Cronbach 0.94	N/A
	The Beliefs about Trauma Scale was created for the current study.	Cronbach 0.81	N/A
	Readiness for Organizational Change Scale (ROC);	Cronbach 0.85	N/A
	Organizational Change Recipients’ Beliefs Scale (OCRBS)	Cronbach 0.84	N/A
Unick et al. (2019) [56]	Level of organizational trauma-informed care was measured using the TICOMETER	Cronbach 0.96	High face and construct validity
Kessler & Isham (2017) [61]	Trauma Informed Care Measure	Initial psychometric properties for the full instrument are unavailable, subscales have alpha coefficients ranging from 0.721 to 0.849	N/A
Hales et al. (2017) [62]	Business Insight survey from Workplace Dynamics.	N/A	N/A
Damien et al. (2017) [63]	Safety Attitudes Questionnaire (SAQ)	Cronbach 0.77	N/A
	Professional Quality of Life (ProQoL).	Cronbach 0.88	N/A

## Data Availability

All data generated as part of this study are included in the article.
